# Microvasculature Features of Vogt-Koyanagi-Harada Disease Revealed by Widefield Swept-Source Optical Coherence Tomography Angiography

**DOI:** 10.3389/fmed.2021.719593

**Published:** 2021-10-14

**Authors:** Xiaoyuan Ye, Haiping Zhang, Peng Xiao, Gengyuan Wang, Xiaoqing Hu, Chun Yan, Fan Li, Yixin Hu, Lishi Su, Jiawen Luo, Jin Yuan, Feng Wen, Wei Chi

**Affiliations:** ^1^State Key Laboratory of Ophthalmology, Zhongshan Ophthalmic Center, Sun Yat-sen University, Guangzhou, China; ^2^Tianjin Aier Eye Hospital, Tianjin, China; ^3^Aier Eye Institute, Changsha, China

**Keywords:** Vogt-Koyanagi-Harada disease, widefield swept-source optical coherence tomography angiography, vascular length density, vascular perfusion density, flow voids

## Abstract

**Background:** Vogt-Koyanagi-Harada (VKH) disease is a multisystem autoimmune disorder which could induce bilateral panuveitis involving the posterior pole and peripheral fundus. Optical coherence tomography angiography (OCTA) provides several advantages over traditional fluorescence angiography for revealing pathological abnormalities of the retinal vasculature. Until recently, however, the OCTA field of view (FOV) was limited to 6 × 6 mm^2^ scans.

**Purpose:** This study examined retinal vasculature and choriocapillaris abnormalities across multiple regions of the retina (15 × 9 mm^2^ wide field, macular, peripapillary regions) among acute and convalescent VKH patients using a novel widefield swept-source OCTA (WSS-OCTA) device and assessed correlations between imaging features and best-corrected visual acuity (BCVA).

**Methods:** Twenty eyes of 13 VHK disease patients in the acute phase, 30 eyes of 17 patients in the convalescent phase, and 30 eyes of 15 healthy controls (HCs) were included in this study. Vascular length density (VLD) in superficial and deep vascular plexuses (SVP, DVP), vascular perfusion density (VPD) in SVP, DVP, and choriocapillaris (CC), and flow voids (FV) in CC were measured across multiple retinal regions via WSS-OCTA (PLEX Elite 9000, Carl Zeiss Meditec Inc., USA) using the 15 × 9 mm^2^ scan pattern centered on the fovea and quantified by ImageJ.

**Results:** Compared to HCs, acute phase VKH patients exhibited significantly reduced SVP-VLD, SVP-VPD, and CC-VPD across multiple retinal regions (all *p* < 0.01). Notably, the FV area was more extensive in VKH patients, especially those in the acute phase (*p* < 0.01). These changes were reversed in the convalescent phase. Stepwise multiple linear regression analysis demonstrated that macular DVP-VLD and macular CC-VPD were the best predictive factors for BCVA in the acute and convalescent VKH groups.

**Conclusion:** The wider field of SS-OCAT provides more comprehensive and detailed images of the microvasculature abnormalities characterizing VKH disease. The quantifiable and layer-specific information from OCTA allows for the identification of sensitive and specific imaging markers for prognosis and treatment guidance, highlighting WSS-OCTA as a promising modality for the clinical management of VKH disease.

## Introduction

Vogt–Koyanagi–Harada (VKH) disease is a multisystemic autoimmune disorder primarily afflicting pigmented tissues. In Asia, VKH disease is a relatively common vision-threatening disorder (1).The classic clinical characteristics are bilateral panuveitis, hypoacusis, meningitis, and cutaneous involvement such as poliosis, vitiligo, and alopecia (2). While the precise mechanisms underlying targeted pigmented tissue attack are still uncertain, it is likely that aberrant T cell-mediated inflammation contributes to disease initiation and maintenance (3, 4). Ophthalmic manifestations could involve the choroidal stroma, retinal pigment epithelium, and outer retina at the posterior pole and peripheral fundus. Inflammation of the choroidal stroma and retinal pigment epithelium layer (RPE) results in a series of changes, including choroidal depigmentation, sunset glow fundus (SGF), subretinal neovascularization, and even exudative retinal detachment (5–7). The clinical course of ophthalmic manifestations can be divided into four stages, prodromal, uveitic, chronic, and chronic recurrent, according to findings from multimodal ocular vascular imaging, traditional indocyanine green angiography (ICGA), fluorescein angiography (FA), and optical coherence tomography (OCT) (5). Injection of fluorescent dye into the circulation reveals characteristic vascular patterns that provide clues to disease progression (8). However, fluorescent imaging modalities (ICGA and FA) do not allow for quantitative analysis of retinal and choroidal blood flow characteristics. Moreover, there are inherent risks from intravenous administration of these dyes.

Optical coherence tomography angiography (OCTA) permits non-invasive, detailed, and depth-resolved imaging of the chorioretinal microvasculature in disease states such as diabetic retinopathy, age-related macular degeneration, retinal vein occlusion, and uveitis (9–15). In addition, several OCTA studies have documented changes in the chorioretinal microvasculature associated with VKH disease (16–24). However, traditional OCTA images provide a limited field-of-view, necessitating montage imaging. Further, the aforementioned OCTA studies analyzed limited vascular features.

The introduction of swept-source technology to OCTA has substantially expanded the potential field of view (FOV), which is particularly advantageous for disorders such as VKH disease afflicting broad regions of the retina. In this study, we enrolled VKH patients in acute and convalescent phases and used the newest PLEX Elite 9000 swept-source OCTA (SS-OCTA) system to capture widefield (15 × 9 mm^2^) OCTA images of the chorioretinal microvasculature in macular and peripapillary regions to provide a more detailed description of disease progression and identify signs indicative of visual dysfunction and recovery.

## Materials and Methods

### Study Population

This cross-sectional, observational study, included 20 eyes of 13 patients with acute VKH disease, 30 eyes of 17 VKH disease patients in the convalescent stage, and 30 eyes of 15 age-matched healthy volunteers without any ophthalmological and/or systemic disorders examined at the Zhongshan Ophthalmic Center from November 2019 to December 2020. Patients were diagnosed based on revised diagnostic criteria established by the First International Workshop on Vogt-Koyanagi-Harada (VKH) disease (25), and grouped according to disease stage. Patients initially diagnosed or receiving systemic corticosteroids for <2 weeks were considered in the acute stage. Among these patients, those with severe exudative retinal detachment, severe anterior chamber inflammation, or vitreous opacity were excluded, and the rest were allocated to the acute stage group. Alternatively, patients receiving systemic corticosteroids for over 3 months and showing no signs of acute ocular inflammation such as choroiditis, serous retinal detachment, disc edema, or exudative retinal detachment were included in the convalescent stage group.

All procedures were performed in compliance with the tenets of the Declaration of Helsinki, and the study was approved by the Ethics Committee of Zhongshan Ophthalmic Center (Guangzhou, China 2019KYPJ127). Written informed consent was obtained from each participant. The following data were collected for all participants: age, sex, best-corrected visual acuity (BCVA) as measured using a Snellen chart, and bilateral intraocular pressure. Patients also received slit-lamp microscopy, indirect fundus ophthalmoscopy, and FA examinations.

### Optical Coherence Tomography Angiography Acquisition

**Figure 1:**
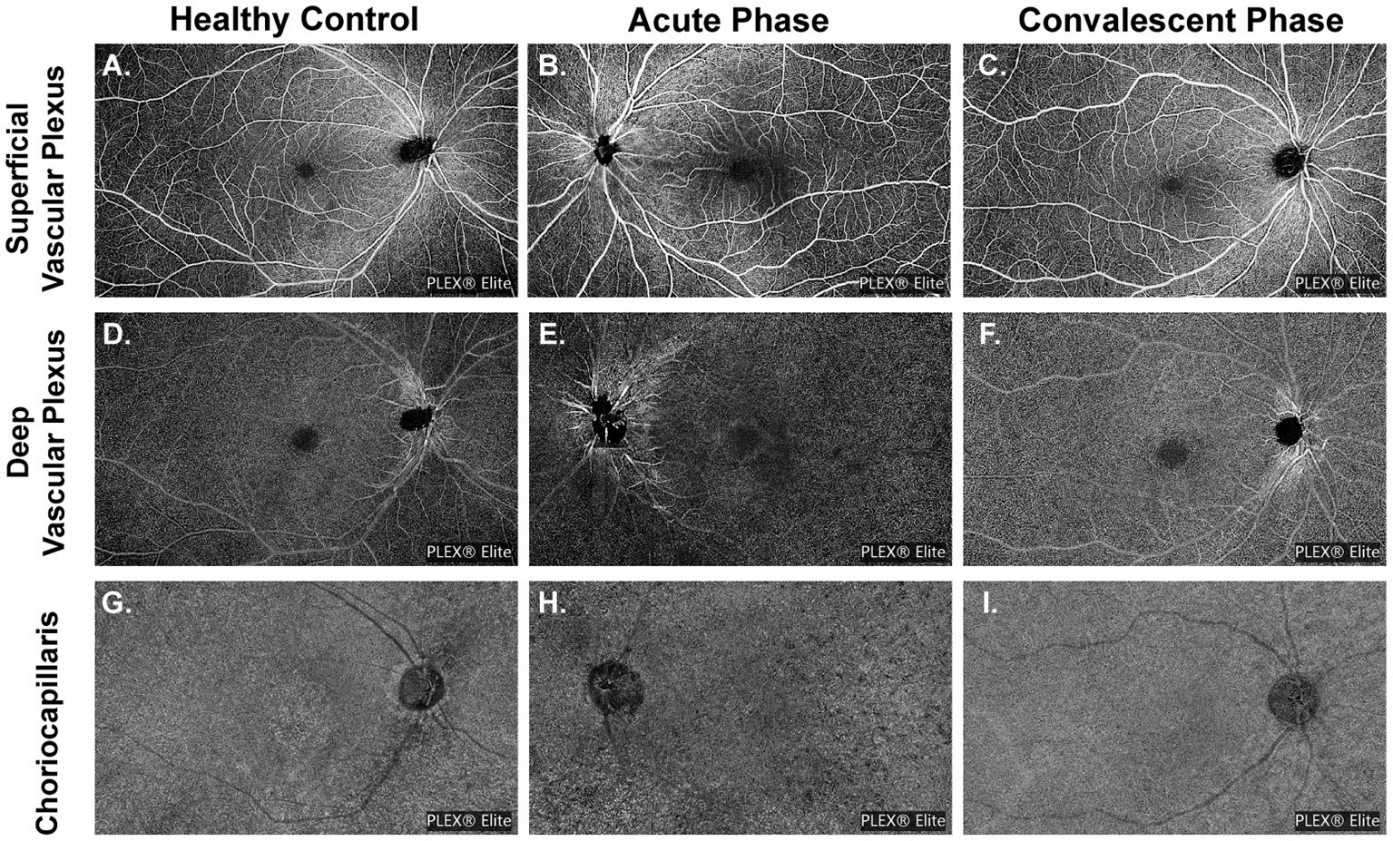
Demonstration of representative images of different slabs in three groups. **(A,D,G)** Slabs are from a healthy volunteer (No. 14, the right eye). **(B,E,H)** Slabs are from a patient with acute VKH disease (No. 12, the left eye). **(C,F,I)** Slabs are from a patient with quiescent VKH disease (No. 17, the right eye).

All widefield swept-source OCTA (WSS-OCTA) images were acquired using a PLEX Elite 9000 SS-OCTA device (Carl Zeiss Meditec Inc., USA) that simultaneously assesses the fundus with a central wavelength of 1,060 nm (1,000–1100 nm full bandwidth) and operates at 100,000 A-scans per second. The system also includes an active eye-tracking system. For each eye, OCTA scans of 15 × 9 mm^2^ centered on the fovea were performed after pupil dilation. All images were collected by the same experienced technician (Hu). Images with either substantial motion artifact or incorrect segmentation were excluded. Representative pictures are presented in Figure 1. To quantify the foveal avascular zone (FAZ), vascular length density (VLD), vascular perfusion density (VPD), and flow voids (FV) across regions and layers, all images were first segmented using built-in software. Segmentation lines defining the inner limiting membrane (ILM), inner plexiform layer (IPL), outer plexiform layer (OPL), and RPE were automatically delineated in each B-scan. Any notable segmentation error was manually corrected. Image slabs were generated to reveal the superficial retinal vascular plexus (SVP) (from the ILM to the outer boundary of IPL), the deep retinal vascular plexus (DVP) (from the outer boundary of IPL to the outer boundary of OPL), and the choriocapillaris (CC) (derived from a 10 μm slab 31–40 μm below the RPE) as previously described (26). Projection artifacts caused by the overlying retinal circulation were removed using built-in software.

### Image Processing and Measurements of the Parameters

**Figure 2:**
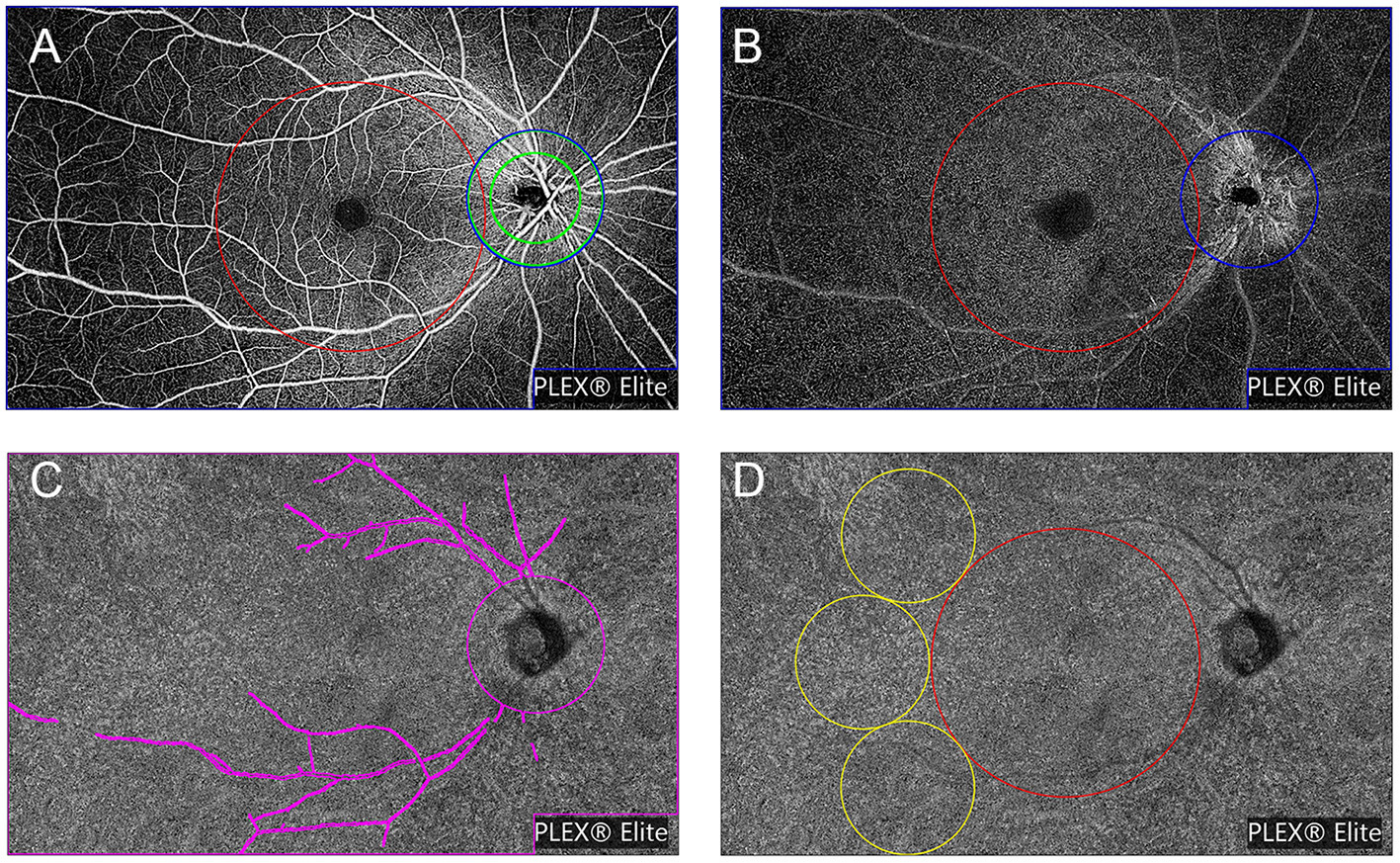
Representation of the regions used to explore OCTA parameters, slabs are from a healthy volunteer (No. 13, the right eye). OCTA parameters were investigated in different regions of the superficial vascular plexus (SVP) slab **(A)** and deep vascular plexus (DVP) slab **(B)**: (1) the macular region (red circle with a diameter of 6 mm centered at the fovea); (2) the peripapillary region (a 500 μm-wide ring of 2 mm inner diameter and 3 mm outer diameter centered at the optic nerve head) (green annulus); and (3) the whole field of view (FOV) with optic papilla area (a 3 mm diameter circle) and the image label “PLEX® Elite” excluded (cropped) (blue polygon shape). **(C)** Representation of whole FOV removing the large retinal vessel mask used to explore OCTA parameters in choriocapillaris slab (shown in magenta). **(D)** Representation of regions to explore flow voids metrics: (1) the macular region (shown in red) and (2) the peripheral region (three 3 mm-diameter circles next to the macular region) (shown in yellow).

All images were checked independently by two trained graders (Ye and Zhang). The OCTA slabs were processed and analyzed according to the procedure used in previous works (16, 27–30). Each OCTA slab was loaded into ImageJ (National Institutes of Health, Bethesda, Maryland, USA; https://imagej.net/Welcome) to measure FAZ, VLD or VPD, and FV parameters as follows. The area and perimeter of the FAZ from the SVP slab were measured by two trained graders by manually outlining the FAZ border in ImageJ and measuring the area and length. The Acircularity Index (AI), defined as the ratio of the FAZ perimeter to the perimeter of a circle with equal area (16), was introduced to describe the acircularity of FAZ shape. “Vascular Perfusion Density” is defined as the fraction of area covered by vessels in an enface slab view (more specifically, the ratio of the vessel area to the total region of interest or ROI), and ranges from 0 (no perfusion) to 1 (fully perfused). The procedure for VPD quantification was as follows. First, images were converted to 8-bit format. Second, the ROI tool in ImageJ was used to select the target regions for analysis, which included the macular region (a 6 mm diameter circle centered at the fovea), the peripapillary area (a 500 μm-wide ring of 2 mm inner diameter and 3 mm outer diameter centered at the optic nerve head), and the whole field of view (whole FOV) with optic papilla area (a 3 mm diameter circle) and the image label “PLEX® Elite” excluded (cropped). Next, images were binarized using commands “Huang's fuzzy” for SVP and DVP slabs or “Phansalkar method” with radius = 15 for CC slabs (27). Finally, the fractional area of vessels within the ROI (or the perfusion density) was measured. To remove large vessel projection artifacts within the 15 × 9 mm^2^ CC slab, we obtained a large retinal vessel mask from the SVP slab by applying “Default” threshold and then used the ImageJ “wand tool” to select the region representing the large vessels. The superficial retinal vessels were successively masked from the CC slab and then the CC slab was binarized using the “Phansalkar method.” Detailed procedures are provided in Figure 2.

“Vascular Length Density” is defined as the vessel length per unit area (31). As far as we know the VPD algorithm is greatly influenced by large blood vessels, and has low sensitivity for detecting capillary changes. Alternatively, the VLD algorithm can more sensitively detect changes in small blood vessels and capillaries. To calculate VLD, we binarized the slab using the “Huang's fuzzy” command and then skeletonized the binary image, followed by measurement of vessel length in the ROI and calculation of VLD as described previously (31, 32). Choriocapillaris flow voids (CC-FV), also termed flow deficit in other studies, is defined as the area lacking flow or with flow below the detectable threshold of OCTA (28, 29). Flow voids were calculated in the macular region (a 6 mm-diameter circle centered at the fovea) and the peripheral region (three 3 mm-diameter circles next to the macular region). Flow void metrics, including total flow void area fraction (ROI area divided by total flow void area), number of FV, and average size of FV, were calculated for FV area > 1,000 μm^2^ (FV1,000) using the “Analyze Particles” function in ImageJ (30).

### Statistical Analysis

All statistical analyses were conducted using SPSS software 22 (SPSS Inc., Chicago, IL). Continuous variables were first tested for normality using the Shapiro–Wilk test and then for homogeneity of variance. Normally distributed variables are presented as mean ± standard deviation (SD) and variables following a skewed distribution as median and 25th percentile to 75th percentile range (P25–P75). Means of parametric datasets were compared by one-way analysis of variance (ANOVA) and non-parametric datasets by the Kruskal–Wallis test, followed by *post-hoc* Bonferroni test or Student *t*-test for pair-wise comparisons. Correlations between parametric variables and logMAR BCVA were analyzed using Pearson Correlation analysis. Stepwise multiple linear regression analysis was run to detect the significant predictors of BCVA. A *p* < 0.05 was considered statistically significant for all tests.

## Results

### Study Population Characteristics

Twenty eyes of 13 patients in the acute phase of VKH disease, 30 eyes of 17 patients in the convalescent phase of VKH disease, and 30 eyes of 15 age-matched healthy controls (HCs) were included in this study. Images of 10 eyes with VKH disease were excluded due to significant motion artifacts or incorrect segmentation. Best-corrected visual acuity of the HCs was not available. The demographic and clinical characteristics of study groups are summarized in Table 1.

**Table 1:** Demographic and clinical characteristics of study groups.

	**Healthy controls**	**Acute phase**	**Convalescent phase**	***p*** **-Value**
Number of eyes/individuals	30/15	20/13	30/17	
Age (years)	34 (26-58)	34 (27-53)	36 (29-43)	0.971^*^
Sex, male/female	5/10	8/5	9/8	0.302^†^
BCVA (LogMAR)	NA	0.60 ± 0.35	0.42 ± 0.28	0.012^‡^
		(0.20–1.30)	(0.00–0.92)	
FAZ (mm^2^)	0.409 ± 0.090	0.334 ± 0.116	0.347 ± 0.110	0.055^§^
	(0.253–0.568)	(0.168–0.543)	(0.184–0.518)	
AI	1.133 ± 0.048	1.122 ± 0.047	1.133 ± 0.034	0.869^§^
	(1.062–1.201)	(1.050–1.182)	(1.080–1.195)	

*BCVA, best-corrected visual acuity; FAZ, foveal avascular zone; AI, acircularity index. Data are presented as Mean ± SD (range) or Median (P25–P75), unless otherwise indicated*.

* *Kruskal–Wallis test*.

† *Chi-square test*.

‡ *Student t-test*.

§ *One-way ANOVA*.

### Widefield OCTA Parameters of Each Group

#### FAZ and AI

The FAZ was smaller in eyes of patients with acute or convalescent VKH disease compared to control eyes, while AI was smaller in acute VKH disease eyes than convalescent VKH disease or control eyes. However, neither difference reached statistical significance (*p* = 0.055 and 0.0869, respectively) (Table 1).

#### Vascular Perfusion Density and Vascular Length Density

**Figure 3:**
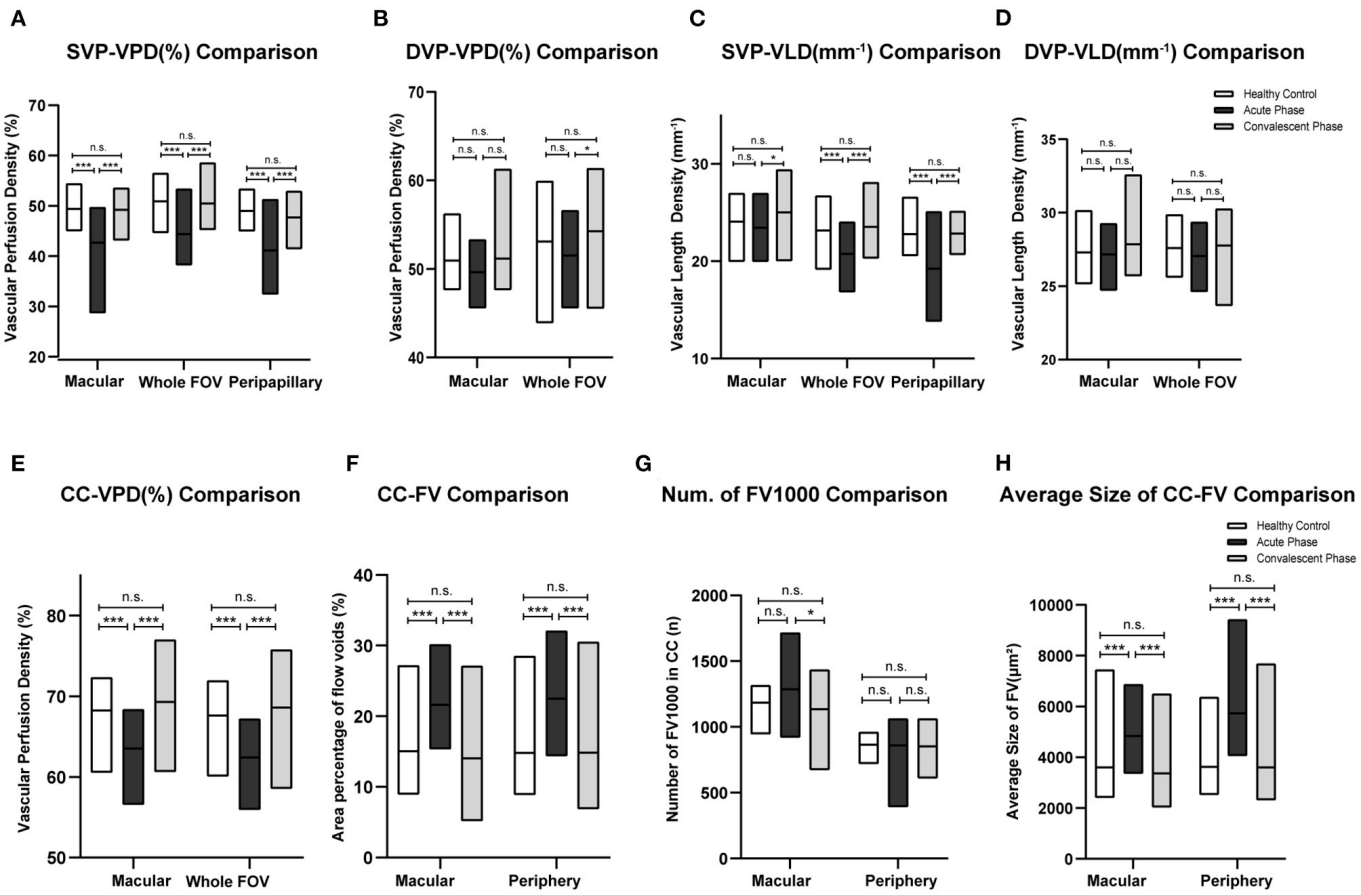
Comparisons of different parameters among three groups in accordance to Tables 2–4. Bar-graphs represent mean (range) **(A–F)** or median (range) **(G,H)**. (^*^p < 0.05; ^***^p < 0.001; n.s., non-significant).

The VPD of the SVP (SVP-VPD) was significantly lower in the peripapillary region, macular region, and whole FOV of acute VKH disease patients compared to HCs and convalescent VKH disease patients (all *p* < 0.001). The same tendency was observed in CC and DVP slabs of the macular region and whole FOV scans. The CC-VPD was also significantly lower in acute VKH disease patients compared to convalescent patients and HCs (both *p* < 0.001). DVP-VPD was also lower in acute phase patients compared to convalescent patients within whole FOV (*p* < 0.05). Compared to HCs, convalescent VKH patients exhibited slightly decreased SVP-VPD and modest increased CC-VPD and DVP-VPD in multiple regions but without statistical significance (Table 2) and (Figures 3A,B). Group differences in VLD roughly mirrored those of VPD, albeit with a few notable differences. In contrast to VPD differences between HCs and convalescent VKH patients, the SVP-VLD of convalescent VKH patients was slightly greater in three regions compared to HCs, but again without reaching statistical significance (Table 3) and (Figures 3C,D).

**Table 2:** The vascular perfusion density (VPD) measurements in different slabs and regions.

**VPD (%)**	**Healthy controls**	**Acute phase**	**Convalescent phase**	***p***	***p** ***^a^**	***p** ***^b^**	***p** ***^c^**
**SVP**							
Macular	49.41 ± 2.55, (48.46–50.36)	42.70 ± 5.14, (40.30–45.11)	49.24 ± 2.39, (48.35–50.13)	<0.001^*^	<0.001	0.907	<0.001
Whole FOV	50.95 ± 3.52, (49.64–52.27)	44.41 ± 4.48, (42.31–46.51)	50.46 ± 3.98, (49.35–51.58)	<0.001^*^	<0.001	1.000	<0.001
Peripapillary	49.04 ± 2.53, (48.10–49.99)	41.15 ± 5.73, (38.47–43.83)	47.72 ± 2.60, (46.75–48.69)	<0.001^†^	<0.001	0.484	<0.001
**DVP**							
Macular	50.93 ± 2.24, (50.10–51.77)	49.63 ± 2.38, (48.52–49.66)	51.17 ± 2.96, (50.10–52.28)	0.101^*^	0.251	1.000	0.123
Whole FOV	53.10 ± 3.59, (51.76–54.44)	51.52 ± 3.38, (49.94–53.10)	54.25 ± 4.32, (52.64–55.86)	0.054^*^	0.474	0.746	0.048
**CC**							
Macular	68.23 ± 2.67, (67.24–69.23)	63.53 ± 2.83, (62.21–64.86)	69.30 ± 4.34, (67.68–70.92)	<0.001^*^	<0.001	0.069	<0.001
Whole FOV	67.59 ± 2.62, (66.61–68.57)	62.42 ± 3.01, (61.01–63.83)	68.61 ± 4.61, (66.89–70.33)	<0.001^*^	<0.001	0.083	<0.001

*VPD, vascular perfusion density; SVP, superficial vascular plexus; DVP, deep vascular plexus; CC, choriocapillaris; FOV, field of view. Data are presented as mean ± SD, (95%CI)*.

* *One-Way ANOVA followed by Bonferroni post-hoc test*.

† *Kruskal-Wallis test followed by Bonferroni post-hoc test*.

p^a^ *: p-value between Healthy Controls and Acute VKH patients*.

p^b^ *: p-value between Healthy Controls and VKH patients in convalescent phase*.

p^c^ *: p-value between Acute VKH patients and VKH patients in convalescent phase*.

**Table 3:** The vascular length density (VLD) measurements in different slabs and regions.

**VLD (mm^−1^)**	**Healthy controls**	**Acute phase**	**Convalescent phase**	***p***	***p** ***^a^**	***p** ***^b^**	***p** ***^c^**
**SVP**							
Macular	24.07 ± 1.91, (23.36–24.79)	21.88 ± 2.54, (20.69–23.07)	24.69 ± 2.04, (23.93–25.45)	0.013^*^	0.692	0.17	0.013
Whole FOV	23.15 ± 1.88, (22.45–23.85)	20.07 ± 1.91, (19.87–21.66)	23.54 ± 1.54, (22.96–24.11)	<0.001^*^	<0.001	1	<0.001
Peripapillary	22.79 ± 1.37, (22.28–23.30)	19.21 ± 2.94, (17.84–20.59)	22.84 ± 1.35, (22.34–23.35)	<0.001^*^	<0.001	1	<0.001
**DVP**							
Macular	27.29 ± 1.13, (26.87–27.71)	27.16 ± 1.30, (26.55–27.76)	27.86 ± 1.68, (27.23–28.48)	0.157^*^	1	0.36	0.263
Whole FOV	27.60 ± 1.01, (27.22–27.97)	27.05 ± 1.35, (26.41–27.68)	27.77 ± 1.71, (27.13–28.41)	0.19^*^	0.52	1	0.224

*VPD, vascular perfusion density; SVP, superficial vascular plexus; DVP, deep vascular plexus; CC, choriocapillaris; FOV, field of view. Data are presented as mean ± SD, (95%CI)*.

* *One-Way ANOVA followed by Bonferroni post-hoc test*.

p^a^ *: p-value between Healthy Controls and Acute VKH patients*.

p^b^ *: p-value between Healthy Controls and VKH patients in convalescent phase*.

p^c^ *: p-value between Acute VKH patients and VKH patients in convalescent phase*.

#### Flow Voids in Choriocapillaris

Acute-stage VKH patients exhibited significantly larger FV area fractions and average FV sizes in both macular and peripheral retinal regions compared to convalescent-stage patients and HCs (all *p* < 0.001). Compared to HCs, convalescent-stage patients exhibited a smaller FV area fraction but larger numbers of FV1,000 (single flow void area >1 000 μm^2^) and larger mean FV1,000 area size in the peripheral region; however, these differences did not reach statistical significance. Details of these comparisons are presented in Table 4 and (Figures 3E-H).

**Table 4:** The flow void parameters in CC slab.

**Flow voids**	**Healthy controls**	**Acute phase**	**Convalescent phase**	***p***	***p** ***^a^**	***p** ***^b^**	***p** ***^c^**
**Macular (6 × 6 mm ^2^)**						
Flow void area (%)	14.80 ± 3.84	21.61 ± 3.80	14.86 ± 5.87	<0.001^*^	<0.001	1.000	<0.001
	(13.54–16.54)	(19.82–23.39)	(11.89–16.19)				
Number of FV1,000 (*n*)	1182.00	1285.00	1211.50	0.007^†^	0.092	0.635	0.004
	(1140.25–1246.25)	(1204.00–1335.00)	(973.50–1273.25)				
Average size (μm^2^)	3332.50	4901.50	3316.00	<0.001^†^	<0.001	1.000	<0.001
	(2911.75–3687.25)	(3998.00–5332.50)	(2466.50–3940.00)				
**Periphery**							
Flow void area (%)	15.04 ± 4.03	22.49 ± 4.49	14.04 ± 5.98	<0.001^*^	<0.001	1.000	<0.001
	(13.37–16.24)	(20.39–24.59)	(12.67–18.84)				
Number of FV1,000 (*n*)	868.00	907.50	901.50	0.513^†^	1.000	1.000	1.000
	(834.75–921.50)	(835.50–964.25)	(710.50–953.25)				
Average size (μm^2^)	3397.00	5607.50	3548.00	<0.001^†^	<0.001	1.000	<0.001
	(3070.75–3948.00)	(4450.50–6625.50)	(2726.25–4208.00)				

*FV1,000, flow void area >1,000 μm^2^. Data are presented as mean ± SD, (95%CI) or median (P25–P75)*.

* *One-Way ANOVA followed by Bonferroni post-hoc test*.

† *Kruskal-Wallis test followed by Bonferroni post-hoc test*.

p^a^ *: p-value between healthy controls and acute VKH patients*.

p^b^ *: p-value between healthy controls and VKH patients in convalescent phase*.

p^c^ *: p-value between acute VKH patients and VKH patients in convalescent phase*.

#### Correlations Between OCTA Parameters and BCVA

To identifying those parameters most strongly associated with BCVA and thus of potential prognostic or diagnostic utility, we conducted Pearson correlation analysis (Table 5). There were strong negative correlations between BCVA and macular DVP-VLD in patients with acute VKH. While at convalescent stage, macular SVP-VPD and parameters in CC have a stronger correlation with BCVA. Stepwise multiple linear regression analysis demonstrated that macular DVP-VLD and macular CC-VPD were the best predictive factors for BCVA in the acute and convalescent VKH groups (Table 6).

**Table 5:** Correlation of best-corrected visual acuity with the parameters of VKH patients in acute phase and convalescent phase.

**BCVA (LogMAR)**	**Acute phase**		**Convalescent phase**	
	***r***	***p*** **-value**	***r***	***p*** **-value**
**SVP–VPD**				
Macular	0.298	0.201	−0.445^*^	0.014
Whole FOV	−0.143	0.549	−0.340	0.066
Peripapillary	0.049	0.837	0.076	0.692
**SVP–VLD**				
Macular	0.381	0.098	−0.210	0.265
Whole FOV	−0.089	0.709	−0.240	0.201
Peripapillary	0.193	0.415	0.275	0.141
**DVP–VPD**				
Macular	−0.367	0.112	0.088	0.644
Whole FOV	−0.132	0.580	−0.353	0.055
**DVP–VLD**				
Macular	−0.500^*^	0.025	0.060	0.752
Whole FOV	−0.154	0.516	−0.303	0.104
**CC–VPD**				
Macular	−0.243	0.301	−0.441^*^	0.015
Whole FOV	−0.370	0.108	−0.373^*^	0.042
**Macular CC–FV**				
Flow void area (%)	0.235	0.319	0.414^*^	0.023
**Peripheral CC–FV**				
Flow void area (%)	0.149	0.532	0.365^*^	0.047

*r, Pearson correlation coefficient. VPD, vascular perfusion density; VLD, vascular length density; SVP, superficial vascular plexus; DVP, deep vascular plexus; CC, choriocapillaris; FV, flow voids; FOV, field of view*.

* *p < 0.05*.

**Table 6:** Stepwise Multiple Linear Regression Analysis for the Predictors of LogMAR BCVA.

**Model summary (acute phase)**	***R*** **^2^**		**Adjusted *R*^2^**	***P*** **-value**
	0.250		0.209	0.025
**Variable**	**Unstandardized coefficients**		**Standardized coefficients**	
	***B***	**Std. error**	**Beta**	
(Constant)	4.675	1.638		0.011
Macular DVP-VLD	−0.148	0.060	−0.500	0.025
**Model summary (convalescent phase)**	***R*** **^2^**		**Adjusted** ***R***^2^	***P*** **-value**
	0.425		0.358	0.030
**Variable**	**Unstandardized coefficients**		**Standardized coefficients**	
	***B***	**Std. error**	**Beta**	
(Constant)	5.309	1.141		0.000
Macular SVP-VPD	−0.033	0.020	−0.261	0.113
Macular CC-VPD	−0.028	0.011	−0.415	0.014
Whole FOV DVP-VPD	−0.025	0.011	−0.356	0.030

*VPD, vascular perfusion density; VLD, vascular length density; SVP, superficial vascular plexus; DVP, deep vascular plexus; CC, choriocapillaris; FV, flow voids; FOV, field of view; R^2^, regression coefficient; Std. error, standard error*.

## Discussion

Vogt-Koyanagi-Harada disease is a multisystemic autoimmune disorder that attacks tissues containing melanin, and thus mainly damages the RPE and choroid in the eyes (2). Acute uveitic phase is characterized by acute, bilateral and diffuse uveitis; hyperemia and edema of the optic disk; choroidal thickening and serous retinal detachment (33, 34). As the disease progresses, there can be signs of SGF or RPE clumping/migration, Dalen-Fuchs nodules or multifocal chorioretinal atrophy (34). Multiple imaging modalities have been used to examine the choroidal features in different stages of VKH disease. On fluorescein fundus angiography (FFA), the retina exhibited disseminated spotted choroidal hyperfluorescence and choroidal hypofluorescence in both acute and chronic uveitic stages (35), while ICGA showed hypofluorescent dark dots during initial acute VKH uveitis episodes that usually resolved after therapy (36). Laser speckle flowgraphy (LSFG), which can non-invasively visualize the hemodynamics of choroidal circulation, revealed inflammation-related impairment in choroidal blood flow velocity at the macula that was again improved by systemic corticosteroid therapy (37).

Optical coherence tomography angiography is a new non-invasive imaging technique that can detect the movement of erythrocytes within blood vessels (9). Compared to spectral-domain OCT (SD-OCT), swept-source OCT (SS-OCT) is able to incorporate longer wavelengths (1,040 to 1,060 nm) and to simultaneously provide images of the vitreous, retina, and choroid (9), yielding high-resolution near-complete images of choroidal anatomy and CC perfusion (38). Therefore, SS-OCTA is a better imaging modality tool for detailed chorioretinal angiography than SD-OCTA.

Flow voids appear as dark foci in the OCTA CC slab and multiple studies have demonstrated that most FV revealed by OCTA correspond to hypofluorescent spots on ICGA images (19, 24, 39–42) and hyporeflective spots on enhanced-depth imaging (EDI)-OCT (43). Aggarwal et al. reported that the CC FV on OCTA images reflected true CC ischemia instead of a shadowing effect from overlying subretinal fluid and RPE detachment as was observed in central serous chorioretinopathy (19). Thus, hypoperfusion spots in CC may be attributed to choroidal granulomas (24, 39) or blood flow perturbance caused by choroidal vessel inflammation (39).

In our study, VKH disease patients demonstrated increased CC-FV and FV average size during the acute phase but not the convalescent phase, consistent with previous studies reporting that systemic corticosteroids can mitigate FV (17, 22, 24, 40). Indeed, the CC-FV observed in convalescent-stage VKH disease patients did not differ significantly from those measured in HCs. Further, the CC-FV area fraction and CC-VPD correlated strongly with logMAR BCVA during the convalescent phase. The CC-VPD was also dramatically reduced during the acute stage but fully restored during the convalescent phase, in contrast to several previous reports (21–23, 44). We speculate that this reflects effective recovery of CC blood flow after appropriate treatment. These positive correlations strongly suggest that choroidal granulomas and persistent inflammation contribute to the vision impairment in VKH disease. Consistent with this notion, Wintergerst et al. reported a case of acute VKH syndrome with massively hypoperfusion in Sattler's layer on OCTA that fully resolved after 4 weeks of treatment concomitant with improved BCVA (20). However, that study was the first to use SS-OCTA for evaluation of CC abnormalities in VKH, so differences in detection device, retinal region, and (or) retinal stratification may have accounted for changes between pre- and post-treatment.

Luo et al. divided acute VKH patients into two subgroups (1 and 2) according to choroid flow area (21), while in the current study we did not observe the subgroup 1 showing significantly enhanced capillary signals, possibly due to the small sample size. Moreover, perfusion density in the CC and flow void size were correlated with visual acuity in our study, suggesting that higher perfusion density [or greater flow area as described by Luo et al. (21)] predict better visual outcome.

In addition to CC, OCTA also allows visualization and quantitative analyses of retinal vessel morphology and perfusion in SVP and DVP slabs. Many researches have revealed a decrease of vascular density when inflammation occurs in fundus, such as Behçet Uveitis (10) and retinal vasculitis (45). Correspondingly, SVP-VPD was markedly reduced in acute VKH disease patients, and a similar trend was also found for the DVP (but did not reach significance). Previous studies have reported similar findings for the SVP, while others have found greater changes in the DVP (16, 23). Different stratification methods may account for these discrepancies. Compared to VPD, the alteration in VLD was relative mild. The SVP-VLD of convalescent VKH disease patients was slightly increased compared to HCs. Though these values were not statistically significant, based on the fact that a minority of VKH patients will develop macular choroidal neovascularization (46), the difference suggests that flow through small blood vessels and capillaries may increase during the convalescent phase as a compensatory mechanism against ischemia caused by inflammation.

A classic manifestation of acute VKH disease revealed by fundus photography is hyperemia and by FFA, hyperfluorescence around the optic disc (35). Surprisingly, however, we found that the peripapillary region VPD was lower among acute VKH disease patients. Therefore, we suggest that inflammation may also affect small vessels near the optic disc. Indeed, peripapillary blood flow is also reduced in patients with optic neuritis (47–49). However, we cannot exclude possible papilledema as eyes afflicted with optic neuritis also showed decreased blood flow (48). Further studies are awaited.

In the current study, logMAR BCVA was strongly correlated with DVP-VLD despite the insignificant changes in DVP among acute VKH disease patients compared to convalescent patients and controls. This may due to enhanced importance of the DVP for outer retina perfusion when the choroid is ischemic (23). Pearson correlation analysis showed parameters in CC slab correlate best with BCVA and stepwise multiple linear regression analysis demonstrated that macular CC-VPD were the best predictive factors for BCVA in the convalescent VKH group. The results indicated a more significant role of CC slab than SVP/DVP in convalescent VKH disease.

Limitations of this study include the modest sample size and grouping based mainly on manifestations in the eye rather than more extensive clinical features. Further, patients received different treatment regimens. Manual correction for inaccuracies of retinochoroidal layer segmentation in images acquired using PLEX 9000 OCTA (performed in this study) may lead to errors in vessel density and perfusion calculations.

To the best of our knowledge, this is the first study to apply a SS-OCTA device to capture widefield 15 × 9 mm^2^ images revealing microvasculature changes associated with VKH disease. The large imaging field avoid potential mistakes of montage pictures thus allow for better simultaneously observation of macular and peripapillary areas in fundus thereby mitigating the many potential errors inherent in analysis of montages. We explore the peripapillary microvasculature for the first time. Besides, we also calculate the total length of blood vessel per unit area (vessel length density) in the retina of VKH patients, which offers a fresh perspective to explore vascular changes in VKH disease. Further studies are needed to confirm our results.

In conclusion, the quantifiable and layer-specific characteristics of wider field SS-OCAT enable better evaluation of the microvasculature features of VKH disease and identification of superior markers for prognosis and treatment guidance.

## Data Availability Statement

The raw data supporting the conclusions of this article will be made available by the authors, without undue reservation.

## Ethics Statement

The studies involving human participants were reviewed and approved by Guangzhou, China 2019KYPJ127, Medical Ethics Committee of Zhongshan Ophthalmic Center, Sun Yat-sen University (Guangzhou, China). Written informed consent to participate in this study was provided by the participants' legal guardian/next of kin.

## Author Contributions

WC, JY, FW, XY, and HZ contributed to conception and design of the study. XH, LS, and JL conducted the OCTA imaging. XY, HZ, PX, and GW processed the images and organized the database. XY, HZ, and FL performed the statistical analysis. XY, HZ, CY, and YH wrote the first draft of the manuscript. JY and WC wrote sections of the manuscript. All authors contributed to manuscript revision, read, and approved the submitted version.

## Funding

This work was supported by National Natural Science Foundation of China (no. 82070950) and Science and Technology Program of Guangzhou of WC (no. 201804010415).

## Conflict of Interest

The authors declare that the research was conducted in the absence of any commercial or financial relationships that could be construed as a potential conflict of interest. The reviewer XL declared a shared affiliation with one of the authors, HZ, to the handling editor at time of review.

## Publisher's Note

All claims expressed in this article are solely those of the authors and do not necessarily represent those of their affiliated organizations, or those of the publisher, the editors and the reviewers. Any product that may be evaluated in this article, or claim that may be made by its manufacturer, is not guaranteed or endorsed by the publisher.
